# Parent-Child Inpatient Treatment in Child and Adolescent Mental Healthcare: Predictors of Child Outcomes

**DOI:** 10.1007/s10578-023-01594-x

**Published:** 2023-08-23

**Authors:** Elena von Wirth, Dieter Breuer, Sabine Schröder, Manfred Döpfner

**Affiliations:** 1 School of Child and Adolescent Cognitive Behavior Therapy (AKiP), Faculty of Medicine and University Hospital Cologne, University of Cologne, Pohligstr. 9, Cologne, 50969 Germany; 2 Department of Clinical Psychology and Psychotherapy for Children and Adolescents, University of Trier, Trier, Germany; 3 Department of Child and Adolescent Psychiatry, Psychosomatics and Psychotherapy, Faculty of Medicine and University Hospital Cologne, University of Cologne, Cologne, Germany

**Keywords:** Inpatient treatment, Mental disorders, Child, Parent-child interaction, Prediction of treatment outcome

## Abstract

Family inpatient units in child and adolescent mental health (CAMH) services engage all admitted family members in the treatment of children’s symptoms. Studies demonstrated improvements in child and family functioning following family inpatient treatment, but evidence regarding predictors of treatment outcome is lacking. We analyzed data of families (*n* = 66) who received a four-week inpatient treatment for families with severe parent-child interaction problems. Hierarchical linear regression analyses revealed that parents who recalled harsher parenting practices of their own fathers reported greater improvements in their children’s externalizing and internalizing problems. Greater improvements in externalizing problems were further predicted by lower parental educational level, less adverse impacts of stressful life events, and less internalizing child problems prior to admission. We therefore conclude that family inpatient treatment was particularly effective for children in families with lower parental education and a history of harsh parenting.

**Supplementary Information:**

The online version contains supplementary material available at 10.1007/s10578-023-01594-x.

## Introduction

It is well-documented that negative parenting practices contribute to the development and maintenance of child and adolescent mental health (CAMH) problems. Harsh and punitive parenting behaviors have been demonstrated to predict emotion regulation difficulties and aggression in children and adolescents [1, 2]. Poor supervision and inconsistent parenting behaviors are associated with attention-deficit/hyperactivity disorder (ADHD) symptoms and oppositional behaviors [3]. Parents who are overprotective may fail to encourage their children to engage in age-appropriate behaviors, thus increasing the risk for developing anxiety symptoms such as separation anxiety and social withdrawal [4, 5]. Positive parenting such as parental warmth, age-appropriate limit setting, and the use of positive reinforcement can, in contrast, serve as a promotive factor that fosters resilience [6, 7].

Given the impact of parenting practices on children’s mental health, parents’ active involvement in their child’s treatment is considered essential to achieving successful outcomes [8]. Behavioral parent trainings (BPTs) that aim at promoting consistent and positive parenting are considered first-line treatments for oppositional-defiant and aggressive behavior problems [9–12] and they have also been shown to be effective in reducing children’s internalizing problems [13, 14]. In addition, meta-analyses demonstrated that parental involvement in their child’s psychotherapeutic treatment improves effect sizes for both externalizing and internalizing outcomes [15–17].

Inpatient psychiatric treatment is usually reserved for children with severe mental health problems. There are several reasons why, for many of these children, admission to a family unit would have clear benefits over admission to a child psychiatric unit without an accompanying parent or caregiver. First, parents of children with severe emotional and behavioral problems often experience elevated levels of strain and emotional distress [18, 19] which adversely affects their children’s well-being through negative effects on parenting [20, 21]. If children and their parents are admitted to a family unit, the clinical team can observe difficult parenting situations directly and help parents to identify individual sources of stress, develop stress management skills, reduce negative parenting practices in stressful situations, and improve positive parenting practices.

Second, it is known that parents’ own mental health problems are strongly associated with their children’s psychopathology [22, 23]. Parental mental health problems can have a disruptive effect on parenting practices and place parents with psychopathology in need of parenting interventions [24]. However, studies have documented that parents with mental health problems tend to attend BPT sessions less frequently [25]. In addition, they are less likely to participate actively, and more likely to drop out of BPT programs compared to parents without mental health problems [26]. Family inpatient treatment may help to overcome these obstacles. The clinical team can help parents to develop an individually tailored parenting plan, and provide practical assistance in the implementation of positive parent-child interaction patterns and parenting practices. In addition, the clinical team can improve parents’ engagement in their child’s treatment by building a positive and trusting parent-therapist alliance, strengthening parents’ motivation, and support parents in seeking help for their own mental health problems.

Third, as pointed out by Cousins and Holmes [27], admitting a child without his or her parents may foster a family’s belief that the child needs to be “fixed” by a clinical team. Admitting an accompanying parent allows clinicians to provide the parent with opportunities to reflect difficult situations and behaviors from a variety of perspectives and to understand how family factors contribute to the development and maintenance of the child’s emotional and behavioral problems. In addition, the clinical team can actively work with the parents, thereby empowering parents to cope with their child’s emotional and behavioral problems and to continue using these coping strategies consistently on discharge.

However, most child psychiatric inpatient units continue to admit children without an accompanying parent or caregiver and parents often attend relatively few family sessions during their child’s inpatient stay [28, 29]. Only few child psychiatric clinics have family units, possibly due to higher costs associated with admitting several family members. In addition, quantitative evidence supporting the effectiveness of family inpatient units in CAMH services is still sparse.

Existing studies have consistently demonstrated improvements in child and family functioning following a family unit stay. For example, a Swedish multicentre study showed that immediately after family inpatient treatment provided by CAMH services half of the families reported a more positive family climate than at the beginning of the treatment [30]. An Australian study evaluated the outcomes of a CAMH family inpatient treatment programme in Melbourne using archival data of 29 families. There were significant improvements in family functioning and in child emotional and behaviour problems following family inpatient treatment [31]. A Norwegian research team showed increases in parental warmth and decreases in mothers’ emotional distress following a family unit stay in a sample of 102 families [32]. A recent re-analysis of the data focused on the effects on child outcomes using a subsample of children (*n* = 50) with available data at admission and at the 3-month follow-up after discharge [33]. There were significant decreases on most Child Behavior Checklist (CBCL [34]) scales, indicating that children showed improvements in a wide array of symptoms and behaviours. A similar finding was reported by Krause et al. [35] who found significant improvements in children’s internalizing and externalizing behaviour problems assessed with the CBCL following family inpatient treatment at a German university hospital in a sample of 60 families.

Since family inpatient treatment is an expensive and restrictive type of care, decisions about admission should be based on patient preferences and on factors predicting treatment outcomes. However, empirical evidence on who is likely to benefit from family inpatient treatment is largely lacking. To our best knowledge, the study by Rimehaug [33] is the only study that has investigated factors associated with response to family inpatient treatment in a CAMH setting. It was found that improvement in parents’ anxiety symptoms, but not depressive symptoms, was correlated with improvement in child symptoms. However, what is needed to guide decisions on whether family inpatient treatment should be considered or not, is information on the predictive value of variables measured before treatment.

Studies on the predictors of outcome of standard child psychiatric inpatient treatment consistently revealed that the child’s age and gender are not related to treatment outcome (e.g., [36–38]). Less favourable treatment outcomes have been found to be predicted by greater mental health problems on admission [38–41]. For example, higher levels of externalizing symptoms such as conduct problems and antisocial behaviours have repeatedly been found to predict less improvement during inpatient treatment [37, 38, 42]. The presence of psychotic symptoms has also been reported to be associated with less favorable outcomes [29, 37, 43]. In addition, it has been found that children and adolescents with lower psychological and social functioning at admission (measured with the Children’s Global Assessment Scale [CGAS]) are more likely to improve their global functioning during psychiatric inpatient treatment [29, 36]. Higher symptom severity, including psychosis, has thus been associated with lower improvement during hospitalization; whereas functional impairment deficits are associated with more potential for improvement during hospitalization.

Family factors have also been found to be important predictors of outcomes of standard child psychiatric inpatient treatment. Several studies revealed that familial risk factors at admission (i.e. unemployment/poverty in the family, marital conflict, disengaged family cohesion, child abuse, involvement of child protection services) predict a less favourable treatment response [37–39, 41, 43, 44]. Blader [45], in contrast, reported that children whose parents reported greater parenting stress at admission showed more improvement in externalizing behavior problems during psychiatric inpatient treatment. Results regarding the predictive value of the family’s socioeconomic status (SES) are also inconsistent. Several studies failed to find a correlation between SES and treatment outcome in adolescent psychiatric inpatients (e.g. [40, 46], but a prospective follow-up study of children discharged from short-term child psychiatric inpatient treatment revealed that a lower level of parental education (which is commonly considered a component of SES) predicted greater improvement in the child’s behavior problems [38].

The treatment offered in family inpatient units differs substantially from standard psychiatric inpatient care. Since parents are admitted along with their child, both are actively involved in the treatment process and parents usually receive intensive treatment that aims at improving parent-child interaction patterns. Consequently, existing evidence on outcome predictors cannot be generalized to family inpatient treatment. To our best knowledge, the present study is the first to investigate which child and family characteristics (measured at admission) predict changes in children’s external and internal problems during family inpatient treatment in a CAMH setting. We used a within-subjects design with participants (*n* = 66, aged 3–10 years) serving as their own controls to evaluate the effectiveness of inpatient treatment for families with severe parent-child interaction problems at the Department of Child and Adolescent Psychiatry, Psychosomatics, and Psychotherapy at the University Hospital Cologne, Germany. We previously reported on the course of outcome measures during the four-week pre-admission period, the four-week inpatient treatment period, and the subsequent four-week follow-up period [47]. Growth curve analyses revealed that standardized measures of children’s internalizing and externalizing behaviour problems were stable during the pre-admission (waiting) period (*d* ≤ 0.1) and improved significantly during the inpatient treatment period (*d* = 0.5–0.6). Significant contrasts indicated that child outcomes improved more during treatment than during the waiting period. In addition, we found that improvements in child outcomes were maintained during the follow-up period (*d* ≤ 0.05). A similar pattern was observed for parent outcomes. Measures of positive parenting practices, parental self-efficacy, parental strains and parental symptoms of depression, anxiety and stress improved significantly more during the treatment period (*d* = 0.4–0.8) than during the pre-admission (waiting) period (*d* ≤ 0.2). Again, benefits stabilized during the follow-up period (*d* ≤ 0.2). We therefore concluded that family inpatient treatment has positive effects on child behavior, parenting and parental mental health.

In the current study, we conducted further analyses of the data to examine predictors of treatment outcomes. We were interested in finding out which families benefit the most from family inpatient treatment and sought to identify variables that predict changes in child emotional and behavioral problems during the four-week treatment period. We considered a large number of variables assessed at admission as potential predictor variables. These include child demographic and behavioral characteristics, family environment, parent psychological characteristics, and parents’ exposure to parenting methods. We sought to identify which predictor variables significantly predict child functioning at discharge (post-treatment) after child functioning at admission (pre-treatment) was considered, thus predicting change in child outcomes during treatment.

## Methods

### Study Design

The main study, which provided data for the current analyses, was conducted as a single-group, within-subjects design (repeated measures) with participants serving as their own controls [47]. There was a four-week pre-admission waiting period, a four-week parent-child inpatient treatment period and a four-week follow-up period. The baseline assessment (T1) was carried out four weeks before admission to parent-child inpatient treatment. The pre-treatment assessment (T2) was done at admission. The post-treatment assessment (T3) was conducted at discharge, immediately after the four-week inpatient treatment period. The follow-up assessment (T4) took place four weeks after discharge. Parents were asked to participate in all measurement occasions. Teachers were asked to complete questionnaires at the T1, the T2 and the T4 assessment. They were not asked to participate in the post-treatment (T3) assessment because the participating children did not attend their regular school during the treatment period. The present analyses are based on data collected at T2 and T3 by a structured parent interview and parent questionnaires. The study was approved by the ethical committee of the Medical Faculty of the University of Cologne.

### Participants

The present analyses are performed in the same sample as the main study [47]. This sample includes 66 children aged 3 to 10 years old (*M* = 6.9, *SD* = 1.7) who had serious behavioural and/or emotional problems and showed severe parent-child interaction problems (clinical judgement of the treatment team). Inclusion criteria were: (i) admission to the parent-child ward of the Department of Child and Adolescent Psychiatry and Psychotherapy at the University Hospital of Cologne between 04/2007 and 04/2009; (ii) children age at least 3 years of age (excluded: *n* = 2); (iii) being an index patient (i.e. not a sibling of a patient; excluded: *n* = 5). Exclusion criteria were: (i) early termination of inpatient treatment (*n* = 1); (ii) inpatient treatment during a period with no data collection due to practical reasons (*n* = 35). Parental verbal informed consent was obtained before admission. A more detailed description of the sample has been previously published [47]. A flow chart is provided in the Electronic Supplement (Figure S1).

The sample is comprised of 17 girls (26%) and 49 boys (74%). The T2 assessment was completed by 66 mothers (64 biological mothers, 2 adoptive mothers, and 2 foster mothers). The T3 assessment was completed by 60 mothers (58 biological mothers and 2 adoptive mothers) and 6 fathers (4 biological fathers and 2 foster fathers). Most children (N = 59, 89%) were of normal intelligence (IQ ≥ 85), seven children (11%) showed below-average cognitive abilities. More than half of the children (60%) had received outpatient psychotherapy of varying intensity prior to admission to the parent-child ward. All participants met criteria for one (*N* = 58) or two (*N* = 8) ICD-10 diagnoses. The most common diagnoses were hyperkinetic disorders with or without comorbid conduct disorder (F90, *N* = 36, 55%), emotional disorders with onset specific to childhood (F93, *N* = 11, 17%), other behavioural and emotional disorders with onset usually occurring in childhood and adolescence (F98, *N* = 8, 12%), pervasive developmental disorders (F84, *N* = 5, 8%), followed by conduct disorders (F91, *N* = 4, 6%), mixed disorders of conduct and emotions (F92, *N* = 4, 6%), reaction to severe stress and adjustment disorders (F43, *N* = 4, 6%), disorders of social functioning with onset specific to childhood and adolescence (F94, *N* = 1, 2%). Seventeen children (26%) used stimulant medication at admission (T2). Four of these (6% of the sample) stopped stimulant treatment during the treatment phase (between T2 and T3). Twelve patients (18%) started stimulant treatment during the treatment phase. At discharge, twenty-five children (38%) were prescribed stimulant medication and 40 children were not medicated (61%). One child (2%) used an antipsychotic drug during the whole study.

### Inpatient Treatment

The parent-child ward of the Department of Child and Adolescent Psychiatry and Psychotherapy at the University Hospital of Cologne provides intensive treatment that aims at improving parent-child interaction patterns, parenting practices, child behaviour and emotional problems, and parental strains. The child and his parents are admitted together and both are actively involved in the treatment process. Up to four families are admitted at a time for a four week period. Both parents can be admitted, but children are usually accompanied by their mother only. All children undergo a structured diagnostic evaluation consisting of psychological and psychiatric assessment, psychological testing (e.g. intelligence), and somatic assessment.

The clinical team consists of a senior clinical psychologist, three educational staff, a child and adolescent psychiatrist, and two child and adolescent psychotherapists in training. They develop an individualized age-appropriate treatment plan for each family that includes child-focused interventions, parent-focused interventions, and parent- and child-focused interventions. The psychosocial interventions are based on evidence-based treatment manuals (e.g. [48].).

Children participate in a daily group training (1.5-2 h/day) that teaches them social and attentional skills (e.g., keeping attention to a task or play activity, social competence training). Individual psychotherapy for the child and pharmacological treatment is offered, as indicated by the child’s age and condition. School-aged children attend the clinic school, which provides education for all children receiving inpatient care at the university hospital. Parents receive group-based behavioural parent training sessions (2 times/week) and individual parent training sessions (2–3 times/week) that target specific behaviour problems of their child. In addition, they are provided with individual psychotherapy as needed (up to 2 or 3 times/week). Parent- and child-focused interventions include dyadic parent-child interaction training consisting of daily positive play time and two weekly coaching sessions. In addition, parents and children participate in weekly parent-child activity groups (e.g., creative activities, excursions) and a weekly therapy session with the whole family (family members that are not admitted to the parent-child ward and/or other professionals involved in the family’s life, such as teachers, youth welfare workers, or psychotherapists may also be invited).

Families spend the weekends at home to generalize new knowledge, skills and behaviour change to the home environment. Special efforts are made to ensure that the families will receive appropriate support after discharge from the parent-child ward. This includes counselling by the clinic’s social service, initiation of youth welfare interventions, initiation of day-treatment programs, initiation of outpatient psychotherapy for the child and / or the parent, as well as support in selection an appropriate school that meets the individual needs of the child.

### Measures

**Child Behaviour Checklist (CBCL).** The CBCL 6–18 [34] (German version [49]) consists of 113 items that describe typical behavioural and emotional problems. Parents complete each item by answering 0 (*Not true*), 1 (*Somewhat or sometimes true*), or 2 (*Very true or often true*). In addition to the Total Problems scale, the CBCL includes two broadband scales (Externalizing, Internalizing) and eight syndrome scales (Withdrawn, Somatic Complaints, Anxious/Depressed, Social Problems, Thought Problems, Attention Problems, Rule-breaking Behaviour, and Aggressive Behaviour). Higher scores refer to greater problems. The German version of the CBCL 6–18 has good reliability (α > 0.93) and validity [49]. Raw scale scores were z-transformed using the sample mean (*M*) and standard deviation (*SD*). The CBCL was completed at pre-treatment and at post-treatment. In the present analyses, pre-treatment CBCL scales (Total scale, Internalizing scale, and Externalizing scale) were used as predictor variables and post-treatment CBCL scales (Total scale, Internalizing scale, and Externalizing scale) were used as outcome variables.

**Structured parent interview.** A structured parent interview was conducted by a member of the clinical team of the parent-child ward at admission (T2). The interview included items pertaining to the family environment (e.g., living conditions, sociodemographic factors), child development, and stressful life events. Thirteen items were considered as potential predictor variables in the present analyses: (1) Parental educational qualification of the better-educated parent (0 = *No school qualifications*, 1–6 different levels of school diplomas in the German school system, higher score = higher level school qualification), (2) Parental professional qualification of the better-qualified parent (0 = *No professional qualification*, 1 = *No recognized professional qualification*, 2 = *Apprenticeship*, 3 = *Commercial / business school*, 4 = *Mastership examination*, 5 = *College*, 6 = *University*), (3) Mother currently working (0 = *No*, 1 = *Yes*), (4) Father currently working (0 = *No*, 1 = *Yes*), (5) Household size (*number of persons living in the household*), (6) Living space size (*size of apartment or house in square meters*), (7) Household income (monthly), (8) Premature birth (0 = *No*, 1 = *Yes*), (9) Birth complications (0 = *No*, 1 = *Yes*), (10) Low birth weight (0 = *No*, 1 = *Yes*), 11) Level of distress during transition to daycare (1 = *Child got used to the group easily*, 2 = *Child had some difficulties getting used to the group*, 3 = *Child had great difficulty getting used to the group*), 12) Number of stressful life events (*Number of events from this list: Family move, Medical Problems, Death of an attachment figure, Parental Separation, Parental Divorce, Remarriage, Separation child – mother, Separation child – father, Financial Problems, Problems with the police, Substance dependencies, Family conflicts, Birth of another child, Occupational stress, Unemployment, Other*), 13) Impact of stressful life events (1 = *Negative*, 2 = *Rather negative*, 3 = *Neutral*, 4 = *Rather positive*, 5 = *Positive*).

**Teacher Report Form (TRF).** The TRF 6–18 [34] (German version [49]) measures teacher-reported behavioural and emotional problems. The 113 items are rated 0 (*Not true*), 1 (*Somewhat or sometimes true*), or 2 (*Very true or often true*). Analogous to the CBCL, the TRF 6–18 yields two broadband syndromes and eight syndrome scales. Higher scores refer to greater problems. Raw scale scores were z-transformed using the sample *M* and *SD*. The German version of the TRF 6–18 has good reliability (α > 0.80) and validity [49].

**ADHD rating scale (FBB-ADHS).** The FBB-ADHS is a parent and teacher rating scale for ADHD symptoms (German: *Fremdbeurteilungsbogen für Aufmerksamkeitsdefizit-/Hyperaktivitätsstörungen*), and is part of the German ICD- and DSM-based Diagnostic System for the Assessment of Mental Disorders in Children and Adolescents (DISYPS-III [50]). The FBB-ADHS contains 20 items that assess the occurrence of ADHD symptoms and are rated on four-point Likert scales ranging from 0 (*Not at all*) to 3 (*Very much*). There are two subscales: Inattention (9 items) and Hyperactivity-Impulsivity (11 items). In addition, a Functional Impairment scale (4 items) assesses impairments associated with ADHD symptoms (overall burden, negative impact on play / school activities, negative impact on relationships with adults, negative impact on peer relationships). Scale scores were z-transformed using the sample *M* and *SD*. Research has shown that the FBB-ADHS is a reliable and valid instrument for parents (α = 0.90 [51]) and teachers (α = 0.96 [52]).

**ODD rating scale (FBB-SSV).** The FBB-SSV is parent and teacher rating scale for symptoms of ODD and CD (German: *Fremdbeurteilungsbogen für Störungen des Sozialverhaltens*) and is also part of the DISYPS-III [50]. The scale contains 25 items that are rated from 0 (*Not at all*) to 3 (*Very much*). Nine items corresponded to the symptom criteria for oppositional defiant disorder (ODD) and 16 items assessed the symptom criteria for conduct disorder (CD). Only the ODD subscale was used due to the participant’s young age. In addition, a Functional Impairment scale (2 items) assesses impairments associated with ODD/CD symptoms (negative impact on social relationships, negative impact on peer interactions). Scale scores were z-transformed using the sample *M* and *SD*. The FBB-SSV has been found to be a reliable and valid instrument for parents (α = 0.89 [53]) and teachers (α = 0.90 [54]).

**Parent Practices Scale (PPS).** The PPS [55] contains 34 items and provides scores for two scales, positive and negative parenting practices. In the present study, only the 13-item positive parenting scale was used. Items measure parents’ patterns of interaction with their children (e.g., “I praise my child”) on a 4-point scale anchored by 0 (“*Never*”) and 3 (“*Almost always*”). Scale scores were z-transformed using the sample *M* and *SD*. A high score refers to positive, reinforcing and supportive parenting behaviour. The German adaptation of the positive parenting practices subscale of the PPS has high internal consistency (α = 0.84) [56].

**Problem Setting and Behaviour Checklist (PSBC).** The PSBC [57] measures parent’s belief in their self-efficacy in solving difficult parenting situations, such as shopping with the child or having visitors arrive. The German adaptation of the PSBC contains 27 items that are rated on 4-point scales anchored by 0 (*Certain I can’t do it*) and 3 (*Certain I can do it*). Scale scores were z-transformed using the sample *M* and *SD*. High scores reflect a high ability to deal with difficult parenting situations. The internal consistency of the German adaptation is high (α = 0.90) [56].

**Self-Efficacy Scale (SEFS).** The SEFS (German: *Fragebogen zur Selbstwirksamkeit in der Erziehung* [58]) is a German adaptation of the Parenting Sense of Competence Scale developed by Johnston and Mash [59] and the Self Efficacy for Parenting Task Index by Coleman and Karraker [60]. The rating scale comprises 15 items that measure parents’ perception of self-efficacy (e.g., “I meet my own personal expectations for expertise in caring for my child”) on a 4-point scale anchored by 0 (*Does not apply to me at all*) and 3 (*Applies to me much, or most of the time*). Scale scores were z-transformed using the sample *M* and *SD*. Higher scores reflect higher self-efficacy. Internal consistency has been shown to be high (α = 0.80) [56].

**Questionnaire on Judging Parental Strains (QJPS).** The QJPS is a 55-item German-language questionnaire that measures the subjective strains of parents of children with ADHD [61]. There are five subscales: Competence and Satisfaction, Solution Orienting, Social Interaction, Partnership and Siblings. Each item (e.g., “My child’s behaviour causes conflict among family members”) is rated on a 4-point scale ranging from 0 (*Does not apply at all / Not distressing*) to 3 (*Very distressing*). Scale scores were z-transformed using the sample *M* and *SD*. High scores reflect high strains. Previous studies reported high internal consistency for parents (α = 0.97) and teachers (α = 0.93) [56].

**Parent Problem Checklist (PPC).** The PPC [62] is a 16–item questionnaire that assesses conflicts between partners over child rearing. Six items explore the extent to which parents disagree over rules and discipline for child misbehaviour, six items assess the occurrence of open conflicts over child-rearing issues, and four items focus on the extent to which parents undermine each other’s relationship with their children. In the German adaptation, the parent is asked to rate for each of the 16 items (e.g. „Parents undermining each other“) the extent to which the issue has been a problem over the past two month on a 4-point scale ranging from 1 (*Not at all*) to 4 (*Very much*). Scale scores were z-transformed using the sample *M* and *SD*. Higher scores reflect higher levels of parental conflict over child-rearing issues. Internal consistency of the German version is high (α = 0.88) [56].

**Depression Anxiety Stress Scale (DASS).** The DASS [63] comprises 42 items that assess symptoms of depression, anxiety and stress in adults (e.g., ‘I could see nothing in the future to be hopeful about’). Parents rate the extent to which they have experienced each symptom over the past week on a 4-point severity/frequency scale anchored by 0 (*Did not apply to me at all*) and 3 (*Applied to me much, or most of the time*). Each of the three subscales (Depression: DASS-DEP, Anxiety: DASS-ANX, Stress: DASS-STR) comprises 14 items. Scale scores were z-transformed using the sample *M* and *SD*. High scores correspond to higher levels of stress, anxiety and / or depression. The internal consistency of the German version was shown to be high for all subscales (α ≥ 0.84) [64].

**Questionnaire of Recalled Parental Rearing Behavior (EMBU**). The Swedish EMBU (‘‘Egna Minnen Beträffande Uppfostran’’ (EMBU) [65] comprises 24 items that measure participants’ memories of their parents’ child-rearing style and behavior. The subscales rejection/punishment, emotional warmth, and control/overprotection relate separately to a person’s mother and father. Each item is rated on a 4-point scale anchored by 1 (“*No, never*”) and 4 (“*Yes*, a*lways*”). Scale scores were z-transformed using the sample *M* and *SD*. The German version of the EMBU has high internal consistency (α ≥ 0.72) [66].

### Data Analysis

The statistical analyses were conducted using the Statistical Package for the Social Sciences, SPSS version 26 (IBM Corporation, Armonk, NY).

**Missing values.** We first checked for missing values in the parent questionnaires. The data set was nearly complete. Only one case (1.5% of 66) had missing pre-treatment data and only two cases (3.0% of 66) had missing post-treatment data. We then checked for missing values in the teacher questionnaires. Eleven cases (17% of 66) had missing TRF data, 12 cases (18% of 66) had missing FBB-ADHS data, and 13 cases (20% of 66) had missing FBB-SSV data at pre-treatment. In addition, four interview items had missing data (Parental professional qualification: *n* = 1 [1.5%], Household size: *n* = 1 [1.5%], Premature birth: *n* = 2 [3.0%], Low birth weight: *n* = 3 [4.6%]). We assumed that missing values in the parent and teacher questionnaires were missing completely at random and imputed missing questionnaire data using the multiple imputation procedure of SPSS. To allow for subsequent analysis including hierarchical linear regression analysis, only one imputation was performed. We did not impute missing interview data.

**Selection of potential predictors for further analyses (pre-analyses)**. We then explored predictors of pre-treatment to post-treatment change using a regression-based model. For each of the three outcome measures (CBCL Total, CBCL Externalizing, CBCL Internalizing) a series of hierarchical linear regression analyses was performed with the post-treatment (T3) CBCL score as the dependent variable. Independent variables were entered block-wise into the regression model (method: ENTER) in the following order: (1) pre-treatment score of the outcome measure; (2) potential predictor variable (pre-treatment assessment). At this step, all pre-treatment measures listed above were considered as potential predictors. The blockwise approach enabled us to identify which predictor variables significantly predict child functioning at discharge (T3) after child functioning at admission (T2) was considered. We did not apply a correction for multiple testing (e.g., Bonferroni), as we aimed to explore these data in order to identify variables for further analyses. Variables that turned out to be significant predictors of change were then used as predictor variables in the subsequent multiple hierarchical linear regression analyses utilizing that outcome measure.

**Multiple, hierarchical linear regression analyses (main analyses)**. Three hierarchical linear regression analyses were conducted with either CBCL Total, CBCL Externalizing, or CBCL Internalizing at post-treatment (T3) as dependent measures. Predictor variables were entered block-wise in the following order: (1) covariate: pre-treatment score of outcome measure (method: ENTER), (2) predictor variables selected based on the results of the pre-analyses (method: STEPWISE).

## Results

### Pre-analyses and Descriptive Statistics

Table S1 (Electronic Supplement) provides the results of hierarchical regression analyses predicting child outcomes (pre-analyses). In each regression analysis, the pre-treatment score of the outcome measure was entered into the regression model first, followed by one potential predictor variable. In these pre-analyses, none of the three outcome measures was significantly predicted by child age, child gender, child medication change during inpatient treatment, interview items pertaining to child development, or teacher ratings of child behavior.

Of the interview items assessing family environment, only parental educational qualification was found to be a significant predictor for one or more outcome measures. In addition, two interview items assessing the impact of life events (level of distress during transition to daycare, impact of stressful life events) were found to be significant predictors for one of the outcomes. Of the parent rating scales assessing child behavior, ratings of Hyperactivity/Impulsivity and ratings of functional impairment due to oppositional behaviors were found to be significant predictors for the outcome CBCL Total. Interestingly, neither parent’s belief in their self-efficacy in parenting tasks (PSBC, SEFS), parental strains (QJPS), parental symptoms of depression, anxiety or stress (DASS), parental conflict over child rearing (PPC) nor parents’ memories of their mothers’ child-rearing style and behaviour (EMBU mother) was found to be a significant predictor for at least one of the outcome measures. Only the PPS (positive parenting practices) and the EMBU scales rejection/punishment (father) and control/overprotection (father) significantly predicted at least one outcome measure. More specifically, the regression analyses predicting CBCL Total scores at post-treatment using CBCL Total scores at pre-treatment as covariate revealed that six variables were significant predictors of change in CBCL Total scores (Parental educational qualifications; FBB-ADHS Hyperactivity-Impulsivity; FBB-SSV Functional Impairment; EMBU fathers‘ rejection/punishment; EMBU fathers‘ control/overprotection).

The regression analyses predicting CBCL Externalizing scores at post-treatment using CBCL Externalizing scores at pre-treatment as covariate revealed that six variables were significant predictors (parental educational qualifications; impact of stressful life events; CBCL Internalizing; PPS; EMBU fathers‘ rejection/punishment).

The regression analyses predicting CBCL Internalizing scores at post-treatment using CBCL Internalizing scores at pre-treatment as covariate revealed that two variables were significant predictors (level of distress during transition to daycare: *p* = .02; EMBU fathers‘ rejection/punishment: *p* = .02).

Table S2 (Electronic Supplement) provides descriptive statistics for the variables selected for further analyses.

#### Multiple Hierarchical Regression Analyses

Table 1 depicts the results of the multiple, hierarchical regression analysis predicting CBCL Total scores (post-treatment). Tests of collinearity indicated that multicollinearity was not a concern (Tolerance ≥ 0.95, VIF ≤ 1.06). In Step 1, the covariate CBCL Total score at pre-treatment was added as a significant predictor of participants’ educational attainment (*F*_(1,64)_ = 13.07, p < .001, Δ*R*^2^ = 0.17). The beta-coefficient was positive (*β* = 0.41), indicating that a higher level of emotional and behavioral problems at pre-treatment was associated with a higher level of emotional and behavioral problems at post-intervention. Step 2 showed that adding parents’ memories of their fathers’ child rearing behavior (EMBU fathers’ rejection/punishment assessed at pre-intervention) as a predictor significantly improved the model (*F*_(1,63)_ = 6.07, *p* = .02, Δ*R*^2^ = 0.07). Parents who reported higher levels of rejection / punishment by their fathers tended to report less emotional and behavioral problems of their child after parent-child inpatient treatment (= more improvement) (*β* = − 0.28). The regression model was further improved by adding the predictor variable Parental educational qualifications in Step 3 (*F*_(1,62)_ = 5.87, *p* = .02, Δ*R*^2^ = 0.07), with lower educational qualifications predicting lower CBCL Total scores at post-treatment (= more improvement) (*β* = 0.26). The final model explained a significant proportion of the variance (31%, adjusted *R*^2^ = 0.28) in children’s emotional and behavioral problems (CBCL Total) at post-treatment (*F*_(3,63)_ = 9.20, *p* < .001).

**Table 1:** Results of hierarchical regression analysis predicting CBCL Total scores (post-treatment)

Predictors (pre-treatment)		*B*	*SE B*	*β*	Δ*R*^*2*^
Step 1					0.17**
	Constant	− 0.04	0.11		
	CBCL Total	0.42	0.12	0.41**	
Step 2					0.07*
	Constant	− 0.01	0.11		
	CBCL Total	0.48	0.12	0.47***	
	EMBU Fathers’ rejection/punishment	− 0.23	0.09	− 0.28*	
Step 3					0.07*
	Constant	− 0.09	0.11		
	CBCL Total	0.51	0.11	0.50***	
	EMBU Fathers’ rejection/punishment	− 0.24	0.09	− 0.29**	
	Parental educational qualifications	0.25	0.10	0.26*	

*Note*. *ΔR*^*2*^ *=* Change in R^2^ associated with the variable(s) entered in each step

CBCL = Child Behavior Checklist, EMBU = Questionnaire of Recalled Parental Rearing Behavior

* *p* < .05. ** *p* < .01

Table 2 depicts the results of the hierarchical regression analysis predicting CBCL Externalizing scores (post-treatment). Tests of collinearity indicated that multicollinearity was not a concern (Tolerance ≥ 0.63, VIF ≤ 1.59). In Step 1, the covariate CBCL Externalizing score at pre-treatment was added as a predictor of participants’ educational attainment (*F*_(1,64)_ = 3.92, p = .052, Δ*R*^2^ = 0.06). The beta-coefficient was positive (*β* = 0.24), indicating that a higher level of externalizing problems at pre-treatment was associated with a higher level of externalizing problems at post-intervention. Step 2 showed that adding CBCL Internalizing pre-intervention scores as a predictor significantly improved the model (*F*_(1,63)_ = 9.78, p = .003, Δ*R*^2^ = 0.13). Parents who reported lower levels of internalizing problems at pre-intervention were more likely to report lower levels of externalizing problems after parent-child inpatient treatment (= more improvement) (*β* = 0.42). The regression model was further improved by adding parents’ memories of their fathers’ child rearing behavior (EMBU fathers’ rejection/punishment assessed at pre-intervention) as a predictor in Step 3 (*F*_(1,62)_ = 10.88, *p* = .002, Δ*R*^2^ = 0.12). Higher levels of rejection / punishment were associated with less externalizing problems after parent-child inpatient treatment (= more improvement) (*β* = − 0.37). In Step 4, the model was significantly improved by adding the predictor variable parental educational qualification (*F*_(1,61)_ = 6.64, *p* = .01, Δ*R*^2^ = 0.07). Lower parental educational level was associated with less externalizing problems at post-treatment (= more improvement) (*β* = 0.27). In Step 5, the model was further improved by adding the predictor variable impact of stressful life events (*F*_(1,60)_ = 5.23, *p* = .03, Δ*R*^2^ = 0.05), with a more negative impact predicting higher CBCL Externalizing scores at post-treatment (= less improvement) (*β* = − 0.26). The final model explained a significant proportion of the variance (42%, adjusted *R*^2^ = 0.38) in children’s externalizing problems (CBCL Externalizing) at post-treatment (*F*_(5,60)_ = 8.85, *p* < .001).

**Table 2:** Results of hierarchical regression analysis predicting CBCL Externalizing scores (post-treatment)

Predictors (pre-treatment)		*B*	*SE B*	*β*	Δ*R*^*2*^
Step 1					0.06
	Constant	− 0.06	0.12		
	CBCL Externalizing	0.25	0.12	0.24	
Step 2					0.13**
	Constant	− 0.05	0.11		
	CBCL Externalizing	0.03	0.14	0.03	
	CBCL Internalizing	0.42	0.14	0.42**	
Step 3					0.12**
	Constant	− 0.01	0.11		
	CBCL Externalizing	0.01	0.13	0.01	
	CBCL Internalizing	0.54	0.13	0.53**	
	EMBU Fathers’ rejection/punishment	− 0.30	0.09	− 0.37**	
Step 4					0.07*
	Constant	− 0.09	0.11		
	CBCL Externalizing	0.06	0.12	0.06	
	CBCL Internalizing	0.54	0.13	0.53**	
	EMBU Fathers’ rejection/punishment	− 0.31	0.09	− 0.38**	
	Parental educational qualifications	0.26	0.10	0.27*	
Step 5					0.05*
	Constant	− 0.07	0.11		
	CBCL Externalizing	0.02	0.12	0.02	
	CBCL Internalizing	0.46	0.13	0.45**	
	EMBU Fathers’ rejection/punishment	-0.35	0.09	− 0.43**	
	Parental educational qualifications	0.24	0.10	0.25*	
	Impact of stressful life events	− 0.25	0.11	− 0.26*	

*Note*. *ΔR*^*2*^ *=* Change in R^2^ associated with the variable(s) entered in each step

CBCL = Child Behavior Checklist, EMBU = Questionnaire of Recalled Parental Rearing Behavior

* *p* < .05. ** *p* < .01

Table 3 depicts the results of the hierarchical regression analysis predicting CBCL Internalizing scores (post-treatment). Tests of collinearity indicated that multicollinearity was not a concern (Tolerance ≥ 0.92, VIF ≤ 1.09). In Step 1, the covariate CBCL Internalizing score at pre-treatment was added as a significant predictor (*F*_(1,64)_ = 8.82, *p* = .004, Δ*R*^2^ = 0.12). The beta-coefficient was positive (*β* = 0.35), indicating that a higher level of internalizing problems at pre-treatment was associated with a higher level of internalizing problems at post-intervention. The regression model was improved by adding the predictor variable EMBU rejection/punishment (father) assessed at pre-intervention in Step 2 (*F*_(1,63)_ = 6.20, *p* = .02, Δ*R*^2^ = 0.08), with higher levels of recalled rejection/punishment predicting lower CBCL Internalizing scores at post-treatment (= more improvement) (*β* = − 0.29). The final model explained a significant proportion of the variance (20%, adjusted *R*^2^ = 0.17) in children’s internalizing problems (CBCL Internalizing) at post-treatment (*F*_(2,63)_ = 7.87, *p* = .001).

**Table 3:** Results of hierarchical regression analysis predicting CBCL Internalizing scores (post-treatment)

Predictors (pre-treatment)		*B*	*SE B*	*β*	Δ*R*^*2*^
Step 1					0.12**
	Constant	− 0.01	0.12		
	CBCL Internalizing	0.36	0.12	0.35**	
Step 2					0.08*
	Constant	0.03	0.11		
	CBCL Internalizing	0.44	0.12	0.43**	
	EMBU Fathers’ rejection/punishment	− 0.24	0.10	− 0.29*	

*Note*. *ΔR*^*2*^ *=* Change in R^2^ associated with the variable(s) entered in each step

CBCL = Child Behavior Checklist, EMBU = Questionnaire of Recalled Parental Rearing Behavior

* *p* < .05. ** *p* < .01

## Discussion

Parenting practices influence children’s behavior and wellbeing and there is reason to assume that, for many children with mental health problems, admission to a family unit would have clear benefits over admission to a child psychiatric unit without an accompanying parent or caregiver. The purpose of the present study was to add to the limited research on the effectiveness of family inpatient units in CAMH services. We were previously able to demonstrate that a four-week parent-child inpatient treatment in a child psychiatric clinic had positive effects on child behavior, parenting, and parental mental health [47]. In the present study, we conducted further analyses of the data. The aim was to identify predictors of change in children’s behavioral and emotional problems during the four-week treatment period.

We conducted three regression analyses to determine which pre-treatment variables best predict the changes in children’s behavioral and emotional problems during family inpatient treatment. For each of the three outcome measures (CBCL Total Problems score, CBCL Externalizing scores, CBCL internalizing scores), a hierarchical regression analysis was conducted with the post-treatment score as dependent variable and the pre-treatment score as a covariate. We then entered pre-treatment child, parent, and family variables as predictor variables into the regression model. The results revealed that changes in CBCL Total scores were best predicted by parents’ (mostly mothers’) memories of their own fathers’ punitive and rejecting parenting practices (β = − 0.29) and parents’ educational qualifications (β = 0.26). More paternal rejection/punishment and less educational attainment predicted greater improvement in children’s emotional and behavioral problems during family inpatient treatment.

Changes in children’s externalizing behavior problems (CBCL Externalizing) were best predicted by children’ internalizing problems (β = 0.45), parents’ memories of their own fathers’ punitive and rejecting parenting practices (β = − 0.43), parental educational qualifications (β = 0.25), and parents’ ratings of the impact of stressful life events on their child’s wellbeing (β = − 0.26). Less parental educational attainment and less internalizing problems of the child on admission predicted greater improvement in children’s externalizing behavior problems. In addition, more paternal rejection/punishment and a more positive impact of stressful life events on the child’s wellbeing on admission were predictive of greater improvement in children’s externalizing behavior problems. We were further able to demonstrate that changes in children’s internalizing symptoms (CBCL Internalizing) were best predicted by parents’ memories of their own fathers’ punitive and rejecting parenting practices (β = − 0.29). More paternal rejection/punishment predicted greater improvements in children’s internalizing symptoms during family inpatient treatment.

Several aspects of these findings deserve discussion. First, parents’ recollections of their own fathers’ punitive and rejecting parenting practices were found to predict treatment-related changes in all three outcomes. A possible explanation is that parenting practices are transmitted across generations [67, 68] and parents who engaged in harsh and punitive parenting behaviors prior to admission may have particularly profited from family inpatient treatment. There is evidence that parents’ memories of the parenting practices they received is linked to the parenting practices they provide to their own children [69]. It is, therefore, likely that parents who describe their fathers’ parenting style as punitive and rejecting engage in negative parenting practices themselves. Negative parenting practices have in turn been found to be linked to children’s emotional and behavioural problems [1, 2, 70]. In addition, reductions in negative and ineffective discipline during BPT have been found to account for child behavior improvement [71–76]. Unfortunately, the present study did not assess negative parenting practices and we are not able to test the hypothesis that the link between parents’ perception of their own fathers’ punitive and rejecting parenting practices and treatment-related improvements in child outcomes can be explained by reductions in negative parenting practices.

Second, educational level was predictive of greater improvements in children’s emotional and behavioral problems. This finding is consistent with earlier work demonstrating that parent’s lower level of education predicted more improvement during short-term child psychiatric inpatient treatment [38]. A possible explanation for the relationship between parent’s educational level and treatment outcome could be that parents with low educational attainments may have engaged in less positive parenting behaviors prior to admission and hence there may have been more scope for improvement in parenting practices. Studies demonstrated that parents that are less educated are less likely to engage in positive parenting practices [67, 77]. In addition, there is evidence of an association between parental education and children’s emotional and behavioral problems [78]. However, in the present study, neither parental reports of positive parenting practices nor parental reports of children’s psychopathology prior to admission turned out to be a significant predictor of change in child outcomes during treatment. In addition, there was no significant correlation between parental educational level and positive parenting practices prior to admission. Therefore, the present data do not support the hypothesis that parents with lower educational attainments engaged in less positive parenting behaviors prior to admission and showed more improvement in parenting practices during inpatient treatment. More research is needed to investigate the process that accounts for greater treatment responses in children from families with lower parental educational level.

Another possible explanation is that the lower educated parents in our sample may have perceived the inpatient treatment as more useful. With regard to parent trainings, research has shown that parents’ subjectively perceived usefulness of the training is associated with change in children’s behavior problems [79]. It is possible that lower educated parents were more satisfied with (and more likely to accept) the treatment components of the parent-child inpatient treatment. They may have perceived the parent training sessions offered during family inpatient training as more useful compared to the higher educated parents in our sample. In addition, children of lower educated parents may have benefitted more from the daily group training and the parent- and child-focused interventions. Since we did not assess subjective evaluations of the usefulness of the parent-child inpatient treatment, further research is needed to clarify the role of parents’ subjective perceptions of the treatment.

Third, we found that the impact of stressful life events on the child’s wellbeing predicted treatment response. Children who experienced more adverse impacts of life events were less likely to show improvement in externalizing behavior problems following family inpatient treatment. The impact of stressful life events was a significant predictor of change even after internalizing and externalizing child symptoms, parents’ memories of their own parents’ childrearing style, and parental educational level were included as predictor variables in the regression model. Interestingly, Boe et al. [80] recently reported that the exposure to stressful life events partly explained the association between SES (measured as combined rating of parents’ education level and economic situation of the family) and children’s mental health problems. Results from the German BELLA cohort-study recently revealed that children of lower educated parents showed more mental health problems in a stressful life situation compared to children of higher educated parents [81]. The finding that family inpatient treatment is less effective in children who were adversely affected by stressful life events indicates that the treatment should focus more on the specific needs of these vulnerable children and their families.

Fourth, we found that more internalizing problems of the child prior to admission predicted less improvement in externalizing problems. This finding may be explained by fact that inpatient treatment was individually tailored to each family’s specific needs. In children with higher levels of internalizing problems and few externalizing symptoms, the interventions focused primarily on reducing internalizing symptoms, such as anxiety or depression. Large improvements on the CBCL Externalizing scale were not to be expected in this group of children.

Finally, it is important to note that, consistent with findings from studies on the predictors of outcome of standard child psychiatric inpatient care (e.g., [36, 38]), child age and gender were not associated with children’s response to treatment. However, we failed to replicate previous work showing less improvement in children with greater mental health problems [29, 37–39]. In the present study, neither the severity of children’s ADHD symptoms nor the severity of children’s ODD symptoms on admission was predictive of changes in children’s behavioral and emotional problems during family inpatient treatment. In addition, the present study failed to demonstrate significant associations between children’s treatment response and the family factors household income, parental conflict, parental strains, parental self-efficacy in solving difficult parenting situations, and parental symptoms of depression, anxiety and stress. This finding was unexpected (see, for example, [37–39, 43, 45]) and demonstrated that evidence on outcome predictors of standard psychiatric inpatient care does not generalize to family inpatient treatment.

Limitations of this study include the lack of an untreated control group, which was considered unethical for these families. Another methodological limitation is the wide array of treatment components which makes it difficult to disentangle the effects of group-based parent training, individual parent training, individual psychotherapy, dyadic parent-child interaction training, children’s daily group training, the weekly parent-child activity groups, the weekly therapy session with the whole family, attendance of the clinic school, and additional individualised services (e.g., initiation of youth welfare). In addition, we relied on unblinded parents’ reports as outcome measures. This may have caused expectancy effects. Parents may have been biased because they were committed to the treatment. However, nonspecific effects (e.g., change in parental expectations) are also important outcomes of the treatment. The use of more objective observational measures, such as videotaped interactions scores by blinded raters, should help to overcome this limitation in future research. Further research is needed to understand the role of subjective evaluations of the usefulness of the different treatment components.

Despite these limitations, we conclude that family inpatient treatment is an effective treatment [47], especially for families with a history of harsh parenting and low parental educational level. Unfortunately, only few child psychiatric hospitals offer family inpatient treatment, while the practice of admitting children with an accompanying parent or caregiver has become the standard practice in medical pediatric treatment (see [27]). The present findings add to the growing literature on the effectiveness of family inpatient treatment in child psychiatric settings and may aid clinical decisions about considering family inpatient treatment as a treatment for child emotional and behavioral problems.

## Summary

To sum up, we found that parent-child inpatient treatment was particularly effective for children whose parents described the parenting style of their own parents as punitive and rejecting. These parents are at risk for engaging in negative parenting practices themselves [69] which may cause or increase emotional or behavioral problems in their children [1, 2]. The present result is promising because it suggests that the intensive treatment provided at the parent-child ward is well suited for families with a history of harsh parenting. In addition, we found that treatment responses were higher in children of lower educated parents compared to children of higher educated parents. This finding is encouraging because previous studies reported that parents with a low SES are less likely to enroll in and to complete parent training programs in outpatient settings [82, 83]. The present finding suggests that parent-child inpatient treatment is successful in fostering parents’ active involvement in their child’s treatment, especially in families with low parental education who are often difficult to keep in treatment. We therefore conclude that admission to the parent-child ward might help to overcome barriers to treatment that low educated parents encounter in outpatient settings.

## Electronic Supplementary Material

Below is the link to the electronic supplementary material.

Supplementary Material 1

## Data Availability

The data that support the findings of this study are not openly available due to reasons of sensitivity and are available from the corresponding author upon reasonable request.
