# Supplementary material for: Parent-Child Inpatient Treatment in Child and Adolescent Mental Healthcare: Predictors of Child Outcomes
Source: Child Psychiatry Hum Dev. 2023 Aug 23;56(3):605–18. doi: 10.1007/s10578-023-01594-x (PMC12095349; doi:10.1007/s10578-023-01594-x)
Supplement: Supplementary file 1 — Supplementary Material 1 [file 10578_2023_1594_MOESM1_ESM.docx]

**Parent-Child Inpatient Treatment in Child and Adolescent Mental Healthcare:**

**Predictors of Child Outcomes**

Elena von Wirth, Dieter Breuer, Sabine Schröder & Manfred Döpfner

**Electronic Supplement**

Figure S1: *Flow of participants through the study*

Children admitted between 04/2007 and 04/2009 (*N* = 114)

Assessment T1 (*n* = 67)

Assessment T4 (*n* = 63)

Assessment T3 (*n* = 66)

Assessment T2 (*n* = 67)

Waiting period

Excluded (*n* = 47)

- Treated during periods with no data collection (*n* = 35)
- Not meeting inclusion criteria:
  - Age less than 3 years (*n* = 2)
  - Sibling (*n* = 5)
- Declined to participate (*n* = 5)

Analyzed (*n* = 66)

- Excluded from analysis:
  - Terminated treatment early (*n* = 1)

Follow-up period

- Drop-out (*n* = 3)

Treatment period

- Drop-out (*n* = 1)

Table S1

*Results of hierarchical regression analyses predicting child outcomes (pre-analyses)*

| Dependent variable  (post-treatment) | | CBCL  Total | | CBCL  Externalizing | | CBCL  Internalizing | |
| --- | --- | --- | --- | --- | --- | --- | --- |
|  |  | *β* | *p* | *β* | *p* | *β* | *p* |
| Step 1: Covariate  (pre-treatment) | |  |  |  |  |  |  |
|  | CBCL Total | .41 | **.001** |  |  |  |  |
|  | CBCL Externalizing |  |  | .24 | .052 |  |  |
|  | CBCL Internalizing |  |  |  |  | .35 | **.004** |
| Step 2: Potential predictor variable  (pre-treatment) | |  |  |  |  |  |  |
| Child age (*n* = 66) | | - .02 | .847 | .03 | .795 | .01 | .916 |
| Child gender (*n* = 66) | | .10 | .403 | .06 | .682 | .01 | .904 |
| Parent interview | |  |  |  |  |  |  |
|  | Parental educational qualification (*n* = 66) | .24 | **.034** | .26 | **.036** | .09 | .459 |
|  | Parental professional qualification (*n* = 65) | .19 | .100 | .22 | .070 | .12 | .332 |
|  | Mother currently working (*n* = 66) | .06 | .618 | .07 | .564 | - .03 | .788 |
|  | Father currently working (*n* = 66) | - .07 | .524 | - .21 | .083 | .06 | .631 |
|  | Household size (*n* = 65) | - .00 | .993 | - .10 | .414 | .04 | .765 |
|  | Living space size (*n* = 66) | .14 | .215 | .16 | .179 | .02 | .892 |
|  | Household income (*n* = 66) | .14 | .216 | .10 | .399 | .04 | .752 |
|  | Premature birth (*n* = 64) | - .04 | .704 | - .14 | .247 | .07 | .567 |
|  | Birth complications (*n* = 66) | - .02 | .833 | - .09 | .448 | .18 | .117 |
|  | Low birth weight (*n* = 63) | - .02 | .843 | - .17 | .187 | .13 | .298 |
|  | Level of distress during transition to daycare (*n* = 66) | .20 | .091 | .24 | .054 | .28 | **.019** |
|  | Number of stressful life events (*n* = 66) | .11 | .372 | .13 | .279 | .06 | .634 |
|  | Impact of stressful life events (*n* = 66) | - .19 | .131 | - .28 | **.028** | .03 | .834 |
| Parent ratings | |  |  |  |  |  |  |
|  | CBCL Externalizing |  |  |  |  | - .07 | .595 |
|  | CBCL Internalizing |  |  | .42 | **.003** |  |  |
|  | FBB-ADHS Inattention (n = 66) | - .10 | .525 | .10 | .511 | - .16 | .225 |
|  | FBB-ADHS Hyperactivity-Impulsivity (*n* = 66) | - .34 | **.018** | - .17 | .312 | - .18 | .130 |
|  | FBB-ADHS Functional Impairment (*n* = 66) | - .24 | .080 | - .18 | .270 | - .16 | .189 |
|  | FBB-SSV Oppositional Defiant (*n* = 66) | - .26 | .119 | - .18 | .418 | - .09 | .482 |
|  | FBB SSV Functional Impairment (*n* = 66) | - .33 | **.027** | - .21 | .226 | - .19 | .139 |
|  | PPS (*n* = 66) | - .20 | .101 | - .26 | **.035** | - .10 | .434 |
|  | PSBC (*n* = 66) | .00 | .959 | - .04 | .791 | - .08 | .517 |
|  | SEFS (*n* = 66) | - .07 | .539 | - .12 | .335 | - .10 | .434 |
|  | QJPS (*n* = 66) | - .11 | .417 | .03 | .871 | - .06 | .615 |
|  | PPC (*n* = 66) | .01 | .917 | - .04 | .747 | .03 | .806 |
|  | DASS Depression (*n* = 66) | .03 | .798 | .13 | .320 | - .06 | .619 |
|  | DASS Anxiety (*n* = 66) | - .00 | .980 | .08 | .525 | - .03 | .835 |
|  | DASS Stress (*n* = 66) | .03 | .841 | .14 | .286 | - .02 | .906 |
|  | EMBU rejection/punishment (mother) (*n* = 66) | - .14 | .231 | - .09 | .463 | - .13 | .309 |
|  | EMBU emotional warmth (mother) (*n* = 66) | - .08 | .471 | - .06 | .609 | .02 | .896 |
|  | EMBU control/overprotection (mother) (*n* = 66) | - .22 | .051 | - .15 | .210 | - .11 | .375 |
|  | EMBU rejection/punishment (father) (*n* = 66) | - .28 | **.017** | - .24 | **.048** | - .29 | **.015** |
|  | EMBU emotional warmth (father) (*n* = 66) | - .00 | .991 | - .02 | .891 | .07 | .571 |
|  | EMBU control/overprotection (father) (*n* = 66) | - .25 | **.029** | - .16 | .190 | - .17 | .159 |
| Teacher ratings | |  |  |  |  |  |  |
|  | TRF Total (n = 66) | - .01 | .961 | .02 | .870 | - .04 | .716 |
|  | TRF Externalizing (n = 66) | - .07 | .581 | - .62 | .658 | - .08 | .511 |
|  | TRF Internalizing (n = 66) | .14 | .250 | .17 | .190 | .09 | .494 |
|  | FBB-ADHS Inattention (n = 66) | .06 | .618 | .11 | .370 | .05 | .663 |
|  | FBB-ADHS Hyperactivity-Impulsivity (n = 66) | - .05 | .685 | .00 | .985 | - .08 | .528 |
|  | FBB-SSV Oppositional Defiant (n = 66) | - .20 | .082 | - .21 | .115 | - .13 | .282 |
| Child medication change during inpatient treatment (*n* = 66) | | .18 | .118 | .08 | .507 | .18 | .128 |

CBCL = Child Behaviour Checklist; FBB-ADHS = ADHD rating scale; FBB-SSV = ODD/CD rating scale, PPS = Parent Practices Scale; PSBC = Problem Setting and Behaviour Checklist; SEFS = Self-Efficacy Scale, QJPS = Questionnaire on Judging Parental Strains; PPC = Parent Problem Checklist; DASS = Depression Anxiety Stress Scale; EMBU = Questionnaire of Recalled Parental Rearing Behavior, TRF = Teacher Report Form

Table S2

*Means (M) and standard deviations (SD) for outcome variables, covariates, and selected predictor variables*

*(n = 66)*

|  |  | *M* | *SD* |
| --- | --- | --- | --- |
| Outcomes variables (post-treatment) | |  |  |
|  | CBCL Total ^a^ | 39.76 | 24.68 |
|  | CBCL Externalizing ^a^ | 16.30 | 10.28 |
|  | CBCL Internalizing ^a^ | 8.53 | 6.84 |
| Covariates (pre-treatment) | |  |  |
|  | CBCL Total ^a^ | 54.58 | 24.75 |
|  | CBCL Externalizing ^a^ | 22.73 | 10.70 |
|  | CBCL Internalizing ^a^ | 12.56 | 7.37 |
| Pre-selected predictor variables (pre-treatment) | |  |  |
|  | Parental educational qualifications (0-6) | 3.71 | 1.73 |
|  | Level of distress during transition to daycare (1-3) | 1.55 | 0.75 |
|  | Impact of stressful life events (1-5) | 2.53 | 1.02 |
|  | FBB-ADHS Hyperactivity-Impulsivity (0-3) ^b^ | 1.49 | 0.85 |
|  | FBB SSV Functional Impairment (0-3) ^b^ | 1.45 | 0.98 |
|  | PPS (0-3) ^b^ | 1.84 | 0.49 |
|  | EMBU rejection/punishment (father) (0-3) ^b^ | 1.72 | 0.85 |
|  | EMBU control/overprotection (father) (0-3) ^b^ | 1.83 | 0.62 |

^a^ Raw score scores (sum of item scores)

^b^ Scale scores (averaged item scores)

CBCL = Child Behaviour Checklist; FBB-ADHS = ADHD rating scale; FBB-SSV = ODD/CD rating scale, PPS = Parent Practices Scale; EMBU = Questionnaire of Recalled Parental Rearing Behavior
